# Supplementary figures and images for: Increased CD14+HLA-DR-/low myeloid-derived suppressor cells can be regarded as a biomarker on disease severity and response to therapy in acute coronary syndrome
Source: PeerJ. 2024 Oct 8;12:e18154. doi: 10.7717/peerj.18154 (PMC11468897; doi:10.7717/peerj.18154)

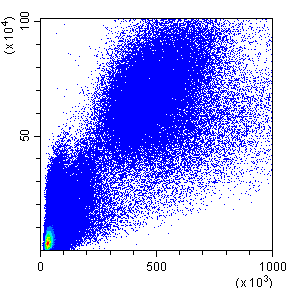

Supplement: Supplemental Information 1 [file peerj-12-18154-s001.tif]

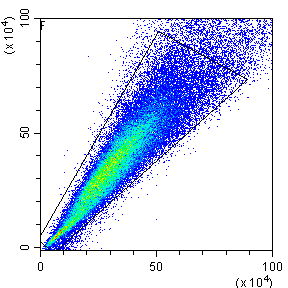

Supplement: Supplemental Information 2 [file peerj-12-18154-s002.tif]

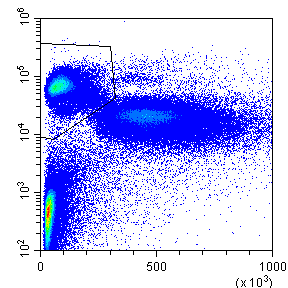

Supplement: Supplemental Information 3 [file peerj-12-18154-s003.tif]

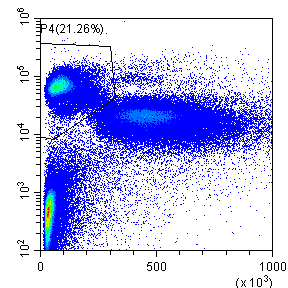

Supplement: Supplemental Information 4 [file peerj-12-18154-s004.tif]

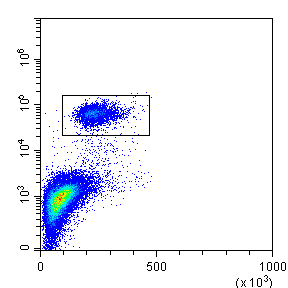

Supplement: Supplemental Information 5 [file peerj-12-18154-s005.tif]

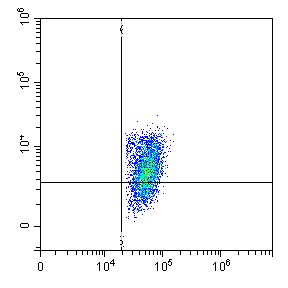

Supplement: Supplemental Information 6 [file peerj-12-18154-s006.tif]

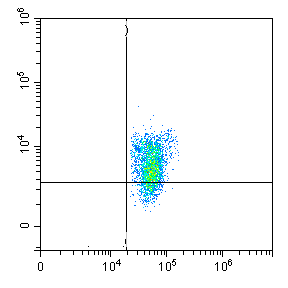

Supplement: Supplemental Information 7 [file peerj-12-18154-s007.tif]

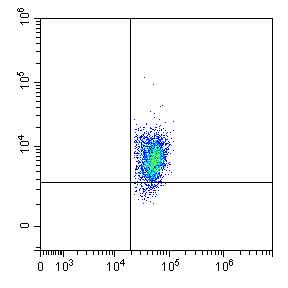

Supplement: Supplemental Information 8 [file peerj-12-18154-s008.tif]

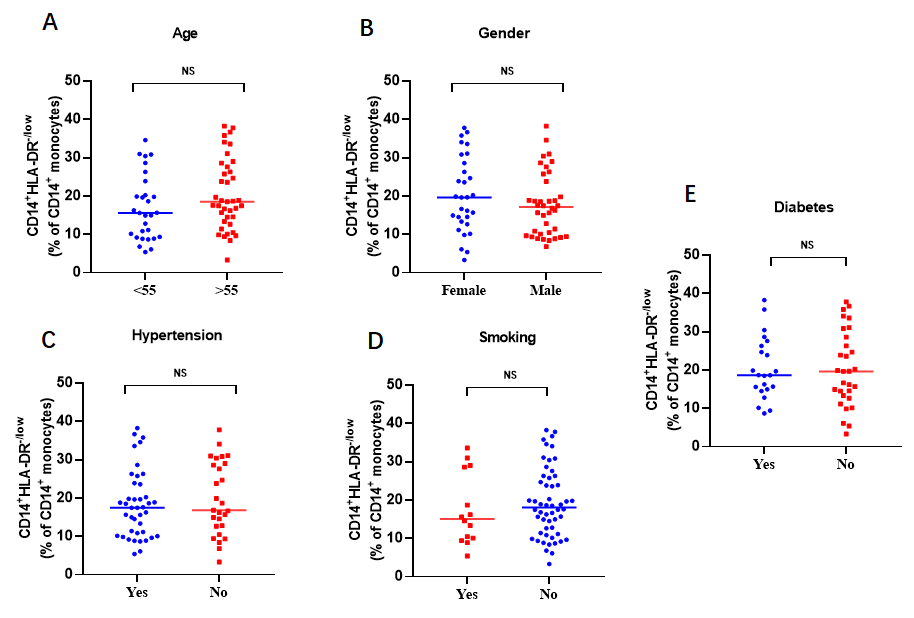

Supplement: Supplemental Information 12 [file peerj-12-18154-s012.tif]
